# Effects of Repetitive Peripheral Magnetic Stimulation through Hand Splint Materials on Induced Movement and Corticospinal Excitability in Healthy Participants

**DOI:** 10.3390/brainsci12020280

**Published:** 2022-02-17

**Authors:** Akihiko Asao, Tomonori Nomura, Kenichi Shibuya

**Affiliations:** 1 Department of Occupational Therapy, Niigata University of Health and Welfare, Niigata 950-3198, Japan; nomura@nuhw.ac.jp; 2 Department of Health and Nutrition, Niigata University of Health and Welfare, Niigata 950-3198, Japan; shibuya@nuhw.ac.jp

**Keywords:** peripheral magnetic stimulation, hand splint, transcranial magnetic stimulation, motor evoked potential, neuromodulation, neurorehabilitation

## Abstract

Repetitive peripheral magnetic stimulation (rPMS) is a non-invasive neuromodulation technique. Magnetic fields induced by rPMS pass through almost all materials, and it has clinical applications for neurorehabilitation. However, the effects of rPMS through clothing and orthosis on induced movement and corticospinal excitability remain unclear. The aim of this study was to determine whether rPMS induces movement and enhances corticospinal excitability through hand splint materials. rPMS was applied directly to the skin (*L*0) and through one (*L*1) or two (*L*2) layers of splint material in 14 healthy participants at 25-Hz, 2-s train per 6 s for a total of 20 min. rPMS was delivered to the forearm with the stimulus intensity set to 1.5-times the train intensity-induced muscle contractions under the *L*0 condition. We recorded induced wrist movements during rPMS and motor-evoked potentials of the extensor carpi radialis pre- and post-application. The results showed that rPMS induced wrist movements in *L*0 and *L*1, and it facilitated corticospinal excitability in *L*0 but not in *L*1 and *L*2. This suggests that rPMS can make electromagnetic induction on periphery even when applied over clothing and orthosis and demonstrates the potential clinical applications of this technique for neurorehabilitation.

## 1. Introduction

Peripheral magnetic stimulation (PMS) is a technique that induces eddy currents, which penetrate the peripheral nerves and muscle spindles, using a time-varying pulsed magnetic field via a coil on the upper and lower extremities as well as the trunk. Repetitive PMS (rPMS) is a novel neuromodulation technique that induces activation of mechanoreceptors of group Ia, Ib, and II nerve fibers during rhythmic contraction and relaxation, similar to muscle vibration [1]. It also induces activation of not only the sensorimotor cortex but also the front-parietal network, including premotor and parietal areas [1,2]. In addition, rPMS modulates the corticospinal excitability and intracortical circuits as well as enhances motor performance in healthy individuals [3,4,5]. Moreover, rPMS is a novel neurorehabilitation method for improving sensorimotor dysfunctions in stroke [1,6,7,8,9,10] and reducing lower back pain [11,12].

rPMS would have greater clinical potentials than peripheral electrical stimulation (PES). The electrical mechanism underlying the stimulation of peripheral nerves and muscle spindles is similar between rPMS and PES [13]. However, there is a salient difference between them. Magnetic stimulation has a magnetic permeability property, and magnetic fields pass through almost all materials without the discomfort of passing through the skin or skull [14,15]. Therefore, rPMS may stimulate peripheral nerves and muscle spindles through not only the skin but also clothing and other materials, whereas PES requires electrodes to be attached to the skin. As a result, rPMS is painless, non-invasive, easy to administer, and penetrates deeper [13]. These are great advantages for the clinical application of neurorehabilitation. While it is theoretically clear that rPMS stimulates muscle spindles and peripheral nerves through clothing or orthosis, this has not yet been investigated experimentally.

Hand splint materials, which are thermoplastics, are used to immobilize, protect, and support the fingers, hands, and forearms during surgery and therapy. They are also used to maintain a fixed functional position in neurorehabilitation [16,17,18,19,20]. In this study, we investigated the effects of rPMS applied over hand splint materials on induced movement and corticospinal excitability in healthy participants. The strength of electromagnetic field induced by the magnetic stimulation coil is inversely proportional to the square of the distance. Accordingly, the change in induced movement during rPMS and corticospinal excitability after rPMS might depend on the distance from the skin to a PMS coil and not on whether there is any hand splint material on the skin. We hypothesized that administering rPMS through the hand splint materials might still be able to induce movements and facilitate corticospinal excitability gradually depending on the layers of hand splint material.

## 2. Materials and Methods

### 2.1. Participants

Fourteen healthy, right-handed adults (7 men and 7 women, mean age ± standard deviation (±SD) = 20.9 ± 0.9 years) participated in this study conducted at the Niigata University of Health and Welfare. The handedness of participants was assessed according to the revised Edinburgh Handedness Inventory [21], with a positive total score reflecting right-handedness (mean score = 92.2 ± 21.9). The participants included in the study had no history of neurological, orthopedic, or psychiatric disease. This study was conducted in accordance with the principles of the Declaration of Helsinki. The study was verbally explained to all participants, and written consent was obtained. This protocol was approved by the Ethics Committee of Niigata University of Health and Welfare (Approval Number: 18129-190117).

### 2.2. Measurement of Induced Movement

**Figure 1:**
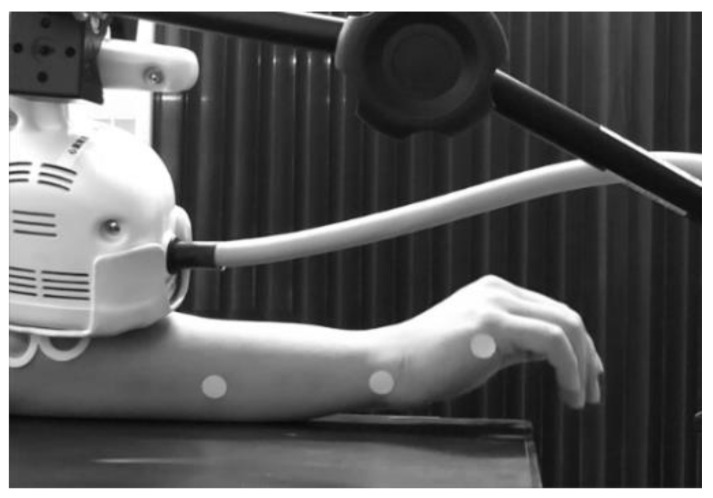
Experimental setup for *L*1 condition. The circular coil connected with the rPMS stimulator was placed on the dorsal side of the forearm. The stimulus coil was placed over the hand splint material. During rPMS, wrist movements were recorded using a home video camera. Three patch seals were attached on the lateral side of the fifth metacarpal phalangeal joint, the ulnar side of the right wrist joint, and the lateral side of the middle of the right forearm for the analysis of the wrist extension angle. *L*1, one splint-material layer; rPMS, repetitive peripheral magnetic stimulation.

The wrist joint movements of each participant induced during rPMS were recorded. Wrist movements were recorded using a home video camera (HDC-TM30, Panasonic, Osaka, Japan) for over 20 min under all experimental conditions, including the rPMS intervention condition. The video camera was set approximately one meter horizontally away from the participant’s right wrist joint for recording its movement induced by rPMS. Three patch seals (diameter: 1 cm) were attached to the right hand and forearm of each participant before rPMS intervention. The first one was on the lateral side of the fifth metacarpal phalangeal joint, the second one was on the ulnar side of the right wrist joint, and the third one was on the lateral side of the middle of the right forearm (Figure 1).

### 2.3. Measurement of Corticospinal Excitability

The motor-evoked potentials (MEPs) were recorded pre- and post-rPMS to assess the corticospinal excitability. Surface electromyography was recorded from the right extensor carpi radialis (ECR) muscle using disposable Ag/AgCl electrodes (Blue Sensor P-00-S; Ambu A/S, Copenhagen, Denmark). The MEP signals were amplified ×100 using a pre-amplification system (A-DL-720-140; 4 Assist, Tokyo, Japan), bandpass-filtered at 5–2000 Hz, digitized at 10 kHz using an A/D converter (PowerLab 8/30; ADInstruments, Dunedin, New Zealand), and stored on a personal computer for offline analysis using LabChart 8.1.8 (ADInstruments). The MEPs were induced by a single-pulsed transcranial magnetic stimulation (TMS). TMS was administered to the scalp through a Figure-eight-coil (internal diameter of each wing: 70 mm) using Magstim 200 (Magstim Co., Whitland, UK). For stimulation of the left primary motor cortex, the coil was placed tangentially at a 45° angle from the midline, with the handle laterally facing the participant’s skull to induce a current from the posterolateral to the anteromedial left brain. Initially, we moved the coil over the left M1, assessed the optimal position (i.e., hot spot) at which maximal MEPs were recorded from ECR, and marked it with a soft-tipped pen. We recorded twelve TMS-induced MEPs before (pre) and immediately after (post) administering the rPMS intervention. TMS was administered at over 10-s intervals. In each experimental session, the TMS intensity was set to induce a peak-to-peak amplitude of approximately 1 mV before rPMS intervention. The TMS intensity was expressed as a percentage of the maximum stimulator output (%MSO).

### 2.4. rPMS

rPMS was delivered to the dorsal side of the right forearm using a Pathleader stimulator (biphasic pulse (width 350 μs)) and a circular coil (outer diameter, 70 mm) (IFG Co., Sendai, Japan). Participants sat in a comfortable chair, held their arm in the prone position on a table, and randomly underwent three different types of rPMS interventions on different days (at least 24 h apart). The intervention conditions were as follows: rPMS using the coil attached directly to the skin, i.e., zero layer of splint material (*L*0); the coil attached to one layer of splint material (*L*1); and the coil attached to two layers of splint material (*L*2). The *L*0 condition was considered a conventional clinical setting of rPMS, in which the coil was placed on the skin, while the *L*1 and *L*2 conditions were considered novel settings of rPMS through hand splint materials. The thermoplastic hand splint material (Rolyan Polyform PAT-A29201, Performance Health Supply Inc., Nottingham, UK; sheet thickness: 3.2 mm; sheet type: plane (no holes in the sheet)) was placed on the bottom surface of the rPMS coil, not on the forearm and hand of the participant (Figure 1); therefore, the right wrist of the participant was not fixed. rPMS was performed at a frequency of 25 Hz, with a stimulus duration of 2 s per train. The stimulus intensity was set at 150% of the minimum intensity that induced visible tetanic muscle contractions of the forearm extensor muscles by an rPMS train with the parameters described above. The rPMS train was delivered every 6 s leading up to a total of 200 trains, lasting 20 min, and 10,000 PMS pulses per experimental condition. The coil was set on the skin of the dorsal side of the right forearm above the ECR muscle using a super clamp, a magic arm, and a stand (Manfrotto, Cassola, Italy) at a position suitable for inducing an extension movement of the wrist in each participant. The location of the coil and the stimulus intensity were assessed during each experiment before the rPMS intervention without using splint materials under all conditions. This meant that the rPMS settings for *L*1 and *L*2 were based on the *L*0 condition.

### 2.5. Data and Statistical Analysis

For the induced movement, two extension angles (degree) were measured using the movement analysis software Kinovea (www.kinovea.org; accessed on 1 January 2022) after all interventions. The first was the minimum angle before an rPMS train, with the rPMS in the OFF state, which was defined as the *Baseline*. The other was the maximum angle during an rPMS train, with the rPMS in the ON state, which was defined as *During*. For each participant, the mean of the *Baseline* and *During* angles was calculated for each experimental intervention. For the corticospinal excitability, the peak-to-peak amplitude (mV) of each MEP was analyzed offline. For each participant, the mean of 10 MEP amplitudes was calculated *Pre*- and *Post*-intervention, after excluding the maximum and minimum amplitudes of each measurement [22,23,24]. The mean stimulus intensities (%MSO) of TMS and rPMS for each experimental condition were calculated. Before performing the statistical analysis, we checked the normality of the distribution of each dataset using the Shapiro–Wilk test. The distribution of the induced movement data was found to not be normal, and thus, we performed a Friedman’s test and calculated Kendall’s W for the effect size. Subsequently, if a significant effect was found, the Wilcoxson signed-rank test was used as the post hoc test, and the r-value was calculated as the effect size. On the other hand, the MEP amplitude data distribution was found to be normal, and thus, we performed two-way (*LAYER: L*0, *L*1, *L*2 × *TIME: Pre*, *Post*) repeated-measures ANOVA and calculated the partial η2 (ηp2) as effect size. Subsequently, if a significant main effect or interaction was found, a paired *t*-test was used for the post hoc test and Cohen’s d was calculated as the effect size. We used one-way (LAYER: *L*0, *L*1, and *L*2) repeated-measures ANOVA to analyze the rPMS intensities. Statistical significance was set at a *p*-value < 0.05. For all post hoc analyses, we used Bonferroni correction. All statistical analyses were performed using R (version 3.4.1; R Foundation for Statistical Computing, Vienna, Austria).

## 3. Results

All participants underwent rPMS under all interventional conditions. No participant reported any adverse effects during or after this study. The mean ± SD of TMS intensities (%MSO) were 63.5 ± 9.4 in *L*0, 66.1 ± 9.7 in *L*1, and 61.3 ± 8.2 in *L*2. The mean ± SD of rPMS intensities (%MSO) were 64.7 ± 8.5 in *L*0, 62.7 ± 7.0 in *L*1, and 64.5 ± 12.0 in *L*2. ANOVA showed no significant effect of rPMS intensity (*p* > 0.05).

### 3.1. Induced Wrist Movements

**Figure 2:**
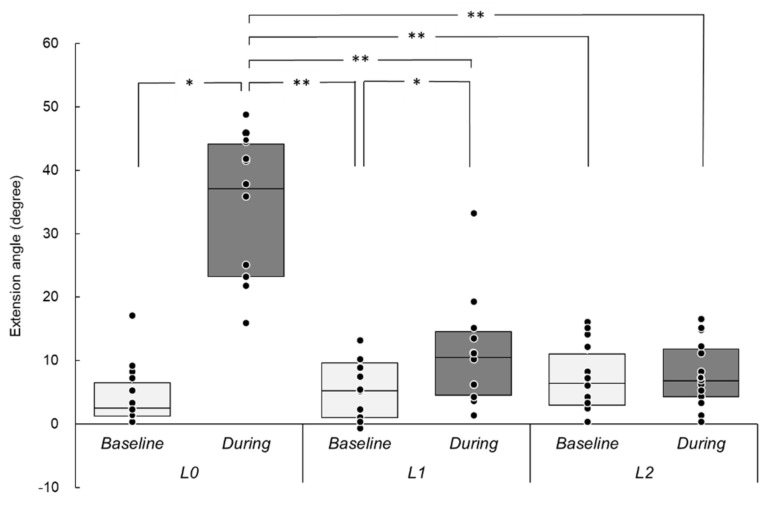
Extension angles under different conditions. The y-axis indicates the wrist extension angle (degree), and the x-axis shows different experimental conditions. *Baseline*, rPMS OFF; *During*, rPMS ON; *L*0, no splint material layer; *L*1, one splint-material layer; *L*2 two splint-material layers. The box plot graphs represent the range from third to first quartile, and the horizontal line in the box represents the median. Each dot represents individual mean value. * indicates a significant difference by pairwise comparison using Wilcoxson signed-rank test adjusted Bonferroni correction. * *p* < 0.05, ** *p* < 0.01. rPMS, repetitive peripheral magnetic stimulation.

Figure 2 shows the extension angles (degree) at *Baseline* and *During* each rPMS intervention. The median (range: min–max) values (degree) were as follows: *Baseline* in *L*0 = 2.5 (0.0–17.0), *During* in *L*0 = 37.1 (15.8–49.2), *Baseline* in *L*1 = 5.3 (−1.0–13.0), *During* in *L*1 = 10.5 (1.1–33.4), *Baseline* in *L*2 = 6.4 (0.0–16.0), and *During* in *L*2 = 6.8 (0.0–16.5). Friedman’s test revealed a significant effect (χ^2^ (5) = 42.19, *p* < 0.001, W = 0.44), and post hoc analyses revealed significant differences between *Baseline* and *During* in *L*0 (Z = 3.30, *p* = 0.02, r = 0.89) and in *L*1 (Z = 3.30, *p* = 0.02, r = 0.89) but not in *L*2 (*p* > 0.05).

### 3.2. MEPs

**Figure 3:**
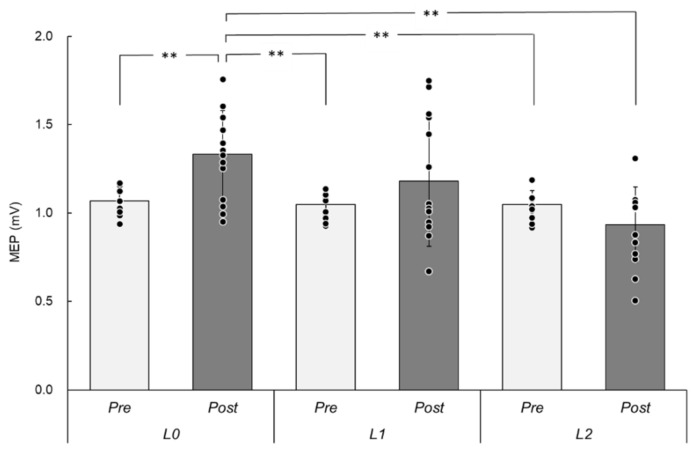
MEPs under different experimental conditions. The y-axis indicates the amplitude of MEPs (mV), and the x-axis shows different experimental conditions. *Pre*, pre-intervention; *Post*, post-intervention; *L*0, no splint material layer; *L*1, one splint-material layer; *L*2 two splint-material layers. The bar graphs and error bars represent mean and standard deviation. Each dot represents individual mean value. * indicates a significant difference by pairwise comparison using paired *t*-test adjusted Bonferroni correction. ** *p* < 0.01. MEP, motor-evoked potential; rPMS, repetitive peripheral magnetic stimulation.

Figure 3 shows the MEP amplitudes (mV) *Pre*- and *Post*-intervention under each rPMS condition. The mean ± SD values were as follows: *Pre* = 1.07 ± 0.08 and *Post* = 1.33 ± 0.25 in *L*0, *Pre* = 1.04 ± 0.07 and *Post* = 1.38 ± 0.37 in *L*1, and *Pre* = 1.05 ± 0.08 and *Post* = 0.93 ± 0.21 in *L*2. A two-way repeated-measures ANOVA revealed a significant main effect of *LAYER* (F (2, 26) = 8.36, *p* = 0.001, ηp2 = 0.39) and an interaction (F (2, 26) = 9.24, *p* = 0.001, ηp2 = 0.42). There was no significant main effect of *TIME* (F (1, 13) = 3.62, *p* = 0.08, ηp2 = 0.22). In pairwise comparisons, there was a significant difference between *Pre*- and *Post*-intervention only in *L*0 (t = 4.70, *p* = 0.006, d = 1.46) but not in the *L*1 and *L*2 conditions (*p* > 0.05).

## 4. Discussion

To the best of our knowledge, this is the first study to investigate the effect of rPMS through hand splint materials on induced movement and corticospinal excitability in healthy participants. Our results show that rPMS induced wrist movement not only when the stimulus coil was placed on the skin but also when applied over one layer of hand splint material. On the other hand, rPMS-induced corticospinal excitability was enhanced only when the stimulus coil was placed on the skin but not when the stimulus coil was placed over hand splint materials.

The kinematic result of this study suggests that rPMS could induce wrist movement through hand splint materials. The wrist movements during rPMS might be dependent on the distance from the stimulus coil to participants’ forearm. The strength of electromagnetic field induced by the magnetic stimulation coil is inversely proportional to the square of the distance (derived from Coulomb’s low, E = kQ/d2). Therefore, the induced movement result indicates that the wrist extensor muscles might be recruited under this electromagnetic property even when there are hand splint materials on the skin. Abe et al. [25] reported that the relation between wrist movements and stimulus intensity during rPMS delivered to the forearm can be fitted with sigmoid curves. Similar to their results, we also found that wrist extension movements induced by rPMS decreased. In the present setting, rPMS was not enough to induce the movement through two layers of hand splint materials. Importantly, these results suggest that rPMS would be able to stimulate muscle valleys and peripheral nerves through not only hand splint materials but also clothing and/or orthosis, while PES cannot.

In the present study, corticospinal excitability did not change after rPMS over hand splint materials. On the other hand, the MEP amplitudes were enhanced after rPMS over the skin, as seen in the *L*0 condition in this study. Previous studies have shown that rPMS activates the cerebral cortex (using recorded somatosensory evoked potentials) [26,27,28]; the front-parietal cerebral network (using positron emission tomography) [1]; and the sensorimotor cortex (using functional magnetic resonance imaging) [2]. In addition, other previous studies have shown that applying rPMS at 25 Hz with intensity above the motor threshold for 20 min facilitated corticospinal excitability of forearm muscles [2,4]. The rPMS settings used in our study were similar to those in the previous studies [2,4], and we showed that rPMS facilitated corticospinal excitability in *L*0. A previous systematic review of PES indicated that the stimulus intensity, especially when it is above the motor threshold, is an important modulator of corticospinal excitability [29]. There might be a common mechanism underlying the induction of cortical plasticity between PMS and PES. Both PMS and PES induce the electrical activation of peripheral nerves and/or muscle spindles, and these afferent inputs might enhance sensorimotor cortex activity [13]. In the present study, while rPMS in *L*0 induced salient movements and enhanced MEPs, rPMS in *L*1 induced small movements but did not enhance MEPs. While this contradicts the findings from PES studies, it is clear that the proprioceptive input induced by rPMS over two layers of hand splint materials is insufficient to facilitate corticospinal excitability. However, rPMS can penetrate almost all materials, and thus, if the intensity is set above the threshold for inducing salient movements, it might induce corticospinal excitability when applied over hand splint materials. In this setting, there might be confounding factors on MEPs, such as the type of stimulator and coil, the distance between stimulus coil and periphery, and the participant’s attention influenced by cutaneous sensation and click sounds due to rPMS. In the future, we need to investigate the effects of rPMS through hand splint materials when the intensity is set above the motor threshold over the hand splint materials experimentally.

The present study demonstrates the potential clinical applications of delivering rPMS through clothing, hand splints, and other orthoses for neurorehabilitation. As a therapeutic intervention, proprioceptive stimulation by rPMS through splint and/or orthosis might support recovery of not only motor functions but also body representation, including body schema and image, in patients with severe sensorimotor dysfunction [30]. Furthermore, it can also be used as an assessment by delivering single-pulsed TMS immediately after rPMS above motor threshold. Whether the MEP would be facilitated or not might act as an indicator that the proprioceptive information by rPMS was incorporated into their body representation [31].

This study has several limitations. First, the number of single-pulsed TMS assessments of corticospinal excitability was smaller than that suggested by previous studies [32,33]. Therefore, we cannot exclude the possibility that the results may be influenced by variability in the MEPs. Second, we investigated the effect of rPMS through hand splint material using only one stimulus parameter setting: frequency, 25 Hz; stimulus train duration, 2 s; intervention time, 20 min; and intensity, 1.5 times of the train intensity-induced muscle contractions under the L0 condition using the present stimulator and coil. It has been reported that the threshold of time-varying biphasic stimuli for neural excitation depends on the pulse duration and the time delay for current reversal [34], which are dependent on coil types, coil direction, and position on inducing peripheral nerve excitability [35,36]. Third, in the present study, the wrist of participants was not fixed. Therefore, the effects of rPMS delivered through clinical hand splint and orthosis that are applied for immobilization remain unclear. Finally, while this sample size was enough for the total number of nine calculated by post hoc power analysis using G*Power software [37], the study cohort was small and consisted only of healthy volunteers. Therefore, investigating patients with sensorimotor dysfunction following stroke, in an experimental setting resembling that of neurorehabilitation facilities, would be clinically more relevant.

## 5. Conclusions

This study demonstrated the effects of rPMS delivered through hand splint materials on induced movement and corticospinal excitability in healthy participants. rPMS through hand splint materials induced wrist movements, and the induced movements decreased with layers of hand splint material. rPMS through hand splint materials did not facilitate corticospinal excitability, while rPMS directly over the skin enhanced MEPs. The present results suggest that rPMS can make electromagnetic induction on periphery even when applied over clothing and orthosis and demonstrate the potential clinical applications of this technique for neurorehabilitation.

## Data Availability

The data that support the findings of the present study are available from the corresponding author upon reasonable request.
